# Medial coronoid prominence and lateral elbow pain: the medial hinge

**DOI:** 10.1016/j.xrrt.2026.100788

**Published:** 2026-06-02

**Authors:** Paolo Arrigoni, Vishal Venkateshmurty Navale, Mikko Raisanen, Alessandro Sorato, Valeria Vismara, Francesco Luceri, Carlo Zaolino, Pietro Simone Randelli

**Affiliations:** a Clinica Ortopedica, Azienda Socio Sanitaria Territoriale Centro Specialistico Ortopedico Traumatologico Gaetano Pini-CTO, Milan, Italy; b Orthopedic Surgery Department, Medicheck Hospital, Faridabad, Haryana, India; c Orthopedic Surgery Department, Faculty of Medicine & Health Technology, Tampere, Finland; d Scuola Di Specializzazione in Ortopedia e Traumatologia Università Degli Studi Di Milano, Milan, Italy; e Laboratory of Applied Biomechanics, Department of Biomedical Sciences for Health, Università Degli Studi Di Milano, Milan, Italy; f U.O.C. 1° Clinica Ortopedica, ASST Centro Specialistico Ortopedico Traumatologico Gaetano Pini-CTO, Milan, Italy; g Research Center for Adult and Pediatric Rheumatic Diseases (RECAP-RD), Department of Biomedical Sciences for Health, Università Degli Studi Di Milano, Milan, Italy

**Keywords:** Lateral elbow pain, Elbow CT arthrography, Coronoid width, Coronoid flange, Medial hinge, Elbow instability, Lateral collateral ligament

## Abstract

**Background:**

Recalcitrant lateral elbow pain is often associated with minor lateral elbow instability, involving laxity of the radial band of the lateral collateral ligament and the annular ligament. To date, the role of coronoid morphology in this pathology has never been studied. This study aims to investigate the potential relationship between lateral elbow pain and specific morphometric measurements of the coronoid process using flexion computed tomography arthrography.

**Methods:**

All elbow flexion computed tomography arthrography from the hospital server was retrospectively analyzed. A total of 133 patients were enrolled and divided into 2 groups: 108 with lateral elbow pain and 25 controls. Coronoid flange and width were measured and compared to the radioulnar articular width in both groups.

**Results:**

No significant morphometric differences in the coronoid process were found between patients with chronic lateral elbow pain and controls.

**Conclusion:**

Other factors, such as capsuloligamentous structures, may have a greater impact on recalcitrant lateral elbow pain compared to the bony morphology of the medial structure of the elbow.

Elbow arthroscopy has revolutionized our understanding of various conditions associated with elbow pain, particularly in cases of recalcitrant lateral elbow pain and minor atraumatic instabilities such as symptomatic minor instability of the lateral elbow and atraumatic posterolateral rotatory posterolateral rotatory instability disease.^2^^,^^5^^,^^14^ Nonetheless, diagnostic elbow arthroscopy remains invasive. Computed tomography (CT) arthrography has emerged as a reliable and accurate method for detailed analysis of elbow pathology, offering high-resolution imaging that can reveal subtle anatomical variations and pathological intraarticular changes.^6^^,^^8^

Static primary stability of the elbow is provided by the ulnohumeral joint, the anterior band of the medial collateral ligament, and by the lateral collateral ligament (LCL) complex. Repetitive stresses in varus and pronation have been shown to lead to attenuation of the LCL structures, mainly the radial band (rLCL) and the annular ligament. Upon a stress in varus, the medial column of the elbow, and more specifically the sublime tubercle, is fundamental for joint stability.^1^^,^^16^ A less prominent sublime tubercle profile provides lower mechanical resistance to varus stresses, especially with the elbow in semiextension (20°-30°).^10^

Morphological variations of the proximal radioulnar joint (PRUJ) have yet to be investigated in a population of patients presenting with recalcitrant lateral elbow pain. Previous research has focused primarily on the lateral structures of the elbow, while the potential influence of medial anatomy on lateral pathology remains poorly understood.^13^ Understanding these relationships could provide valuable insights into the pathogenesis of lateral elbow pain and guide treatment strategies.^7^^,^^17^

The primary aim of this study is to investigate the correlation between the presence of lateral elbow pain and specific CT arthrography measurements of the sublime tubercle process, comparing these findings with control subjects.

## Materials and methods

This level III retrospective cohort study analyzed adult patients who underwent flexion CT arthrography for elbow evaluation between January 2018 and January 2025 at a single institution. The study protocol was approved by the institutional ethic committee, and all patients provided written informed consent before participation.

All adult patients with lateral elbow pain lasting for at least 6 months, positive to SALT (Supination and Antero-Lateral pain Test) and PEPPER (Posterior Elbow Pain by Palpation-Extension of the Radiocapitellar joint) tests,^3^ and availability of flexion CT arthrography were included as cases. In the control group were included patients who underwent the exam for other clinical reasons (loose body identification, medial collateral ligament tears, or elongation), without a history of lateral elbow pain. Patients with conditions affecting the elbow’s bony architecture were excluded to maintain study homogeneity (history of fracture dislocation, primary osteoarthritis, post-traumatic osteoarthritis, coronoid dysplasia).

The exam was performed using a 64-slice CT scanner (GE Revolution EVO) following ultrasound-guided intra-articular injection of iodinated contrast agent via a trans-tricipital approach.^13^^,^^20^ Patients were positioned prone with the affected upper limb elevated and the elbow flexed at 45° to optimize imaging conditions by relaxing the anterior capsule, annular ligament, and radial band of LCL (rLCL) while preventing olecranon engagement in the fossa. CT slice thickness was set at 0.625 mm, with an effective radiation dose of approximately 0.2 mSv. The entire procedure typically required 15-20 minutes.

**Figure 1:**
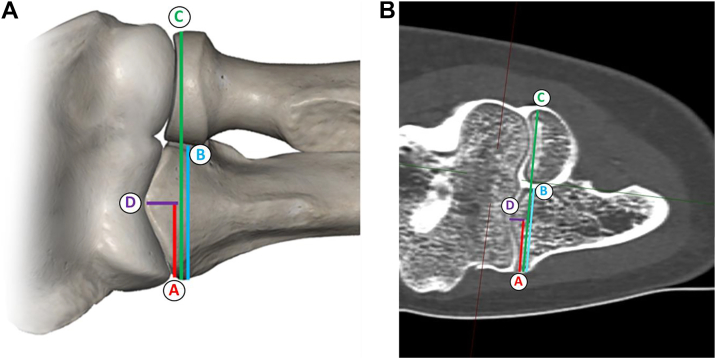
Graphical representation (**A**) and flexion computed tomography arthrography scan (**B**) of the assessments of proximal ulna and radius in coronal plane (mm): *A*, medial coronoid flange; *B*, total coronoid width; *C*, radioulnar articular width; *D*, maximum articular height at the coronoid midpoint.

**Figure 2:**
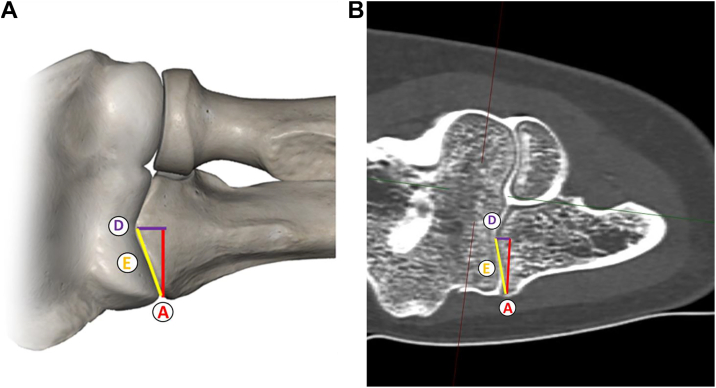
Graphical representation (**A**) and flexion computed tomography arthrography scan (**B**) of the assessments of proximal ulna and radius in coronal plane (mm): *A*, medial coronoid flange; *D*, maximum articular height at the coronoid midpoint; *E*, the hypotenuse between the segments A and D.

All measurements were performed by an orthopedic surgeon looking at the coronal series showing maximum sublime tubercle process width and radial head profile. All measurements were normalized using the radioulnar articular width as a reference (C) to account for individual anatomical variations. Total ulnar width (B) was identified from the medial border of the sublime tubercle to the PRUJ. Medial ulnar flange (A) was measured from the most medial portion of the coronoid process up to a perpendicular line passing through the tip of the coronoid, defined as the maximum articular height at the coronoid midpoint (D). The hypotenuse between the segments A and D was identified by letter E (Figs. 1 and 2). An independent orthopedic surgeon, who was blinded to the clinical status of the patient examined, performed all measurements.

Statistical analysis included normality assessment using the Shapiro-Wilk test, with an alpha risk of 5% (α = 0.05). Correlation analyses were performed using Spearman's method, with *P* values < .05 considered significant. Differences between groups were evaluated with Student's *t*-test, chi-squared test, or Fisher's exact test, according to the characteristics of data distributions. Finally, Spearman's method was used to assess the correlations.

A sample size calculation was performed before data collection. Considering a clinically significant difference of 10% in normalized measurements between groups, with an alpha of 0.05 and a power of 80%, a minimum of 82 patients was required in the lateral pain group. For the control group, while maintaining the same statistical parameters, a minimum of 20 available subjects was deemed sufficient for comparison. The final sample size of 108 patients in the lateral pain group and 25 controls exceeded these requirements, ensuring adequate statistical power for the analysis.

## Results

Overall, this study involved 133 patients: 108 cases, affected by recalcitrant lateral elbow pain, and 25 controls. Demographic analysis revealed comparable characteristics among groups, with a mean age of 51 years in the lateral pain group (74 males, 68.5%) and 49 years in the control group (19 males, 76.0%).

Initial analysis of the radioulnar articular width (C), used for normalization, showed comparable values between groups. The lateral pain group had a mean width of 47.5 mm (36.8-60.7), while the control group showed a mean of 48.7 mm (37.4-58.9). After normalization, we analyzed 4 key measurements.

**Figure 3:**
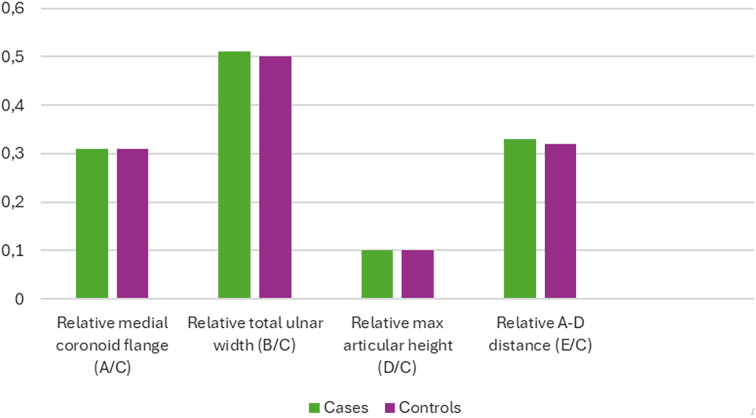
All measurements are normalized to the radioulnar articular width (C). Values shown inside bars represent group means. No significant differences were found between groups (*P* > .05).

The medial coronoid flange (A) showed an identical mean of 0.31 in both groups, with ranges of 0.26-0.36 in the cases group and 0.27-0.36 in controls (*P* = .81). The total coronoid width (B) was slightly larger in the cases group (mean: 0.51, range: 0.44-0.60) compared to controls (mean: 0.50, range: 0.45-0.57), though this difference was not statistically significant (*P* = .79). The maximum articular height at the coronoid midpoint (D) was consistent between groups, with both showing a mean of 0.10, albeit with slightly different ranges (cases: 0.06-0.15; controls: 0.05-0.14; *P* = .75). Finally, the correlation distance between points A and D (hypotenuse, E) was also similar between groups, with a mean of 0.33 in cases and 0.32 in controls (case range: 0.28-0.38; control range: 0.28-0.37; *P* = .85) (Table I and Fig. 3).

**Table I:** Subgroup analysis of assessed measurements.

Variables	Cases (n = 108)	Controls (n = 25)	*P* value
Mean age, yr	51	49	>.05
Gender, males, n (%)	74 (68.5%)	19 (76.0%)	>.05
Raw radioulnar articular width (C)	47.5 (36.8 - 60.7)	48.7 (37.4 - 58.9)	>.05
Relative medial coronoid flange (A/C)	0.31 (0.26 - 0.36)	0.31 (0.27 - 0.36)	>.05 (.81)
Relative total ulnar width (B/C)	0.51 (0.44 - 0.60)	0.50 (0.45 - 0.57)	>.05 (.79)
Relative max articular height (D/C)	0.10 (0.06 - 0.15)	0.10 (0.05 - 0.14)	>.05 (.75)
Relative A-D distance (E/C)	0.33 (0.28 - 0.38)	0.32 (0.28 - 0.37)	>.05 (.85)

Normalized measurements are presented as mean (range). All measurements (A-E) were normalized to the radioulnar articular width (C).

## Discussion

This study represents the first comprehensive morphometric analysis of the proximal ulnar shape in patients with lateral elbow pain using flexion CT arthrography. Our findings suggest that medial sublime tubercle morphology, as measured by various standardized parameters, does not differ significantly between patients with lateral elbow pain and controls.

The possible protective role of a congruent coronoid process in the setting of lateral elbow pain and minor elbow instability has not yet been investigated. This study suggests that the medial extension of the tubercle does not seem to be involved in the pathogenesis of recalcitrant elbow pain. Indeed, no difference was found between patients with recalcitrant lateral elbow pain and controls. Although the coronoid process is not directly implicated as a primary source of pain, coronoid injury or dysplasia may exacerbate the biomechanical conditions that cause lateral pain. In addition, this is a preliminary analysis, and further assessment will be needed to validate our research.

The coronoid process is critical for elbow stability, especially against varus stress. Several cadaveric studies show that a deficiency of more than 50% of the coronoid process is associated with increased instability.^10^ In the acute trauma setting, the anteromedial coronoid opposes medial buckling and should always be restored to avoid early osteoarthrosis development due to persistent elbow instability.^9^ In these patients, ulnar morphology has been investigated in terms of proximal ulna dorsal angulation and of coronoid opening angle.^4^^,^^12^^,^^18^ Both contribute significantly to elbow stability and can directly influence the lateral compartment. Alterations in the proximal ulna dorsal angulation angle, such as those observed in fractures or malunions, can lead to joint misalignments, which can increase the load on surrounding structures, including those in the lateral compartment, contributing to joint instability and functional overload. In addition, a reduction in coronoid opening angle reflects a proportional decrease in the height of the coronoid process, which has direct implications for joint stability.^15^^,^^19^ In the chronic setting, biomechanical changes could increase the load on lateral ligamentous structures, such as the radial LCL, contributing to functional overload, the onset of chronic pain, and functional limitations.

The possible protective role of the coronoid process has not yet been investigated in the setting of recalcitrant lateral elbow pain. Repetitive stress in varus and pronation has been shown to lead to elongation and degeneration of the rLCL and annular ligament, giving rise in the long run to intra-articular lesions.^2^ Kim et al recently described a pattern of recurrent osteoarthritic changes in the elbow joint (ventral top of capitellum, distal end of the lateral trochlear ridge, and beveled rim of the radial head) and investigated the possibility of an anterolateral rotatory instability as the primum movens of the pathology.^11^ In this model, the PRUJ internally rotates as a unit about the long axis of the ulna beyond the normal physiologic limit relative to the humeral articular surface, with the elbow in midflexion, around 90° upon full pronation of the arm. Among possible contributing factors, among possible contributing factors, both the LCL elongation and the loss of function of the proximal medial collateral ligament were highlighted.

Flexion CT arthrography is a reliable technique in the evaluation of recalcitrant lateral elbow pain. Due to its exceptional spatial resolution and ability to visualize fine details of intra-articular structures, this technique has proven particularly effective in detecting pathologies often not evident with other diagnostic methods.^8^ This imaging technique is also very accurate in evaluating complex bone structures such as the coronoid process. Thanks to the possibility of performing high-resolution multiplanar reconstructions, this technique allows the coronoid process to be analyzed with millimeter precision, detecting deformities, erosions, or fractures that may contribute to joint instability, which could be a source of lateral pain.^8^

In recent years, there has been a great focus on the ligamentous structures involved in the pathogenesis of recalcitrant lateral elbow pain, but little has been said about the bony architecture, which could favor its development. Our study suggests that the coronoid process and, in particular, the medial extension of the sublime tubercle might not be involved. When examining the specific measurements, all normalized parameters showed remarkably similar values between groups. The medial coronoid flange, total coronoid width, maximum articular height, and correlation distance measurements demonstrated no statistically significant differences. A more congruent joint, which could be seen as an increase in the medial coronoid flange or the maximal articular height, was not found in control cases. The difference between cases and controls should be sought somewhere else, focusing more on the soft tissues rather than on bony components. The consistency across multiple parameters strengthens our conclusion that coronoid process morphology may not play a substantial role in the development of lateral elbow pain.

The strength of our study lies in its standardized measurement protocol and the use of normalization to account for individual anatomical variations. The normalization process, using the radioulnar articular width as a reference, allowed for reliable comparisons between patients of different sizes and builds. Additionally, our comprehensive approach to measuring multiple aspects of coronoid morphology provides a thorough assessment of this anatomical structure's potential relationship with lateral elbow pain.

However, our study has several limitations that warrant discussion. First, an important bias is that the measurements were carried out by a single examiner and, thereby, intercorrelation coefficient could not be calculated. The control subjects were not healthy individuals: we deemed it reasonable to choose patients who underwent CT arthrography for other unrelated pathologies as controls, since flexion CT arthrography can be a painful medical exam and would require the healthy controls to undergo ionizing radiation. In addition, there is a notable difference in gender distribution between groups, with males comprising 68.5% of the lateral pain group and 76.0% of the control group. While this difference might introduce potential bias, the small size of the control group (n = 25) makes it difficult to determine if this disparity significantly impacts our findings. Future studies with larger, gender-balanced cohorts may provide additional insights into potential gender-specific variations in coronoid morphology.

The retrospective nature of our study represents another limitation, as it inherently introduces the possibility of selection bias. Although our sample size exceeded the minimum requirements based on power analysis, the relatively small control group might limit the generalizability of our findings.

## Conclusion

Our analysis revealed no significant correlations between coronoid process morphometry and the presence of lateral elbow pain. These findings suggest that the etiology of lateral elbow pain likely involves factors beyond coronoid process anatomy, supporting recent theories about the multifactorial nature of lateral elbow pathology. Future prospective studies with larger sample sizes may be warranted to further investigate potential anatomical contributors to lateral elbow pain.

## Disclaimers

Funding: No funding was disclosed by the authors.

Conflicts of interest: The authors, their immediate families, and any research foundations with which they are affiliated have not received any financial payments or other benefits from any commercial entity related to the subject of this article.
